# The second victim phenomenon among midwives in Austria (SeViD-A3): A cross-sectional study

**DOI:** 10.18332/ejm/206922

**Published:** 2025-07-16

**Authors:** Victoria Klemm, Eva Potura, Sabine Fuerst, Hannah Roesner, Reinhard Strametz

**Affiliations:** 1 Wiesbaden Institute for Healthcare Economics and Patient Safety, RheinMain University of Applied Sciences, Wiesbaden, Germany; 2 The Second Victim Association Austria, Vienna, Austria

**Keywords:** midwives, occupational health, psychological distress, patient safety, emotional distress, second victim

## Abstract

**INTRODUCTION:**

Midwives frequently encounter adverse events, potentially leading to the Second Victim Phenomenon (SVP), a condition marked by emotional distress, self-doubt, and psychological symptoms. This study investigates the prevalence, key triggers, symptom severity, and support needs of midwives in Austria affected by SVP.

**METHODS:**

A cross-sectional survey was conducted among Austrian midwives (n=487) using the SeViD-questionnaire. Descriptive statistics were applied to assess SVP prevalence, symptoms, and support measures. Binary logistic regression analyses examined predictors of SVP occurrence and symptom severity, while the Mann-Whitney U test compared support preferences between affected and non-affected midwives.

**RESULTS:**

SVP prevalence was 94.3%, with self-doubt (52.5%), guilt (47.0%), and flashbacks (41.8%) as the most pronounced symptoms. Work experience and workload did not predict SVP occurrence. However, events involving patient harm (OR=1.92; 95% CI: 1.11–3.31, p=0.02) and higher neuroticism scores (OR=1.62; 95% CI: 1.24–2.11, p<0.01) significantly predicted high symptom severity. Affected midwives rated professional counseling as less helpful than non-affected peers (p=0.03, r=0.02).

**CONCLUSIONS:**

The high prevalence of SVP underscores the need for comprehensive, accessible support for Austrian midwives. Peer support programs should be central, alongside preventive and reactive measures. Support must be inclusive of self-employed midwives. Targeted interventions can enhance mental well-being, improve patient safety, and strengthen healthcare quality.

## INTRODUCTION

Midwives can be involved in all stages of pregnancy, childbirth, and postpartum care. In Austria, midwifery is regulated by the Midwifery Act (Hebammengesetz), which defines education, licensure, and scope of practice. Midwives work in hospitals and private practice and are essential providers throughout the perinatal period. Throughout these processes, unexpected adverse events, such as arrested labor, extensive bleeding, or even maternal or fetal death, can occur^1^. Studies on the prevalence of such events have found that at least 71% of surveyed midwives reported experiencing at least one adverse event^2,3^. Adverse events perceived as traumatizing by midwives are mainly characterized by five features: they are unexpected and sudden, extremely severe, involve multiple complications, are difficult to control, and result in negative or lasting outcomes^4^. Following these experiences, midwives may become Second Victims (SVs) – healthcare workers involved in an unanticipated adverse event who are negatively impacted by the experience^5^. The Second Victim Phenomenon (SVP) affects not only SVs themselves but also their future patients and healthcare organizations. SVs often experience guilt, frustration, emotional distress, and fear of professional consequences^6,7^. Long-term symptoms, such as shame, guilt, and loss of confidence, can resemble those of post-traumatic stress disorder^8^. SVP can also impact patient care, as SVs may engage in defensive medical practices, potentially resulting in the omission of necessary treatments or the performance of unnecessary procedures^9^. Additionally, healthcare organizations may be affected by increased absenteeism, presenteeism, turnover rates, and staff leaving the profession entirely^10^.

SVP is common among healthcare workers, with prevalence estimates ranging from 10% to 89% across various medical professions^7,11,12^. Earlier studies reported lower prevalence rates between 10% and 43%^7^, whereas more recent studies indicate higher rates^11,12^. Since 2021, the SeViD-studies (Second Victims in German-speaking countries) have been conducted among different medical professionals in Germany and Austria^11-15^. These studies employ the SeViD-questionnaire to examine the prevalence of SVP, triggering key events, reception of support after an event, time of recovery, symptoms, and preferred support measures^16^. So far, the SVP has been investigated among young physicians in internal medicine, nurses, and emergency medical services physicians in Germany^13-15^. In Austria, SVP among pediatricians and nurses has been analyzed^11,12^. The Austrian studies have revealed higher prevalence rates among the two studied professions in both professions comparison to their German counterparts.

This study is the third to use the SeViD-questionnaire in Austria and, to our knowledge, the first to investigate SVP among midwives in German-speaking countries. Given the limited research on SVP specifically within the midwifery profession and the absence of studies focusing on this issue in German-speaking contexts, the aim of this study was to gain a deeper understanding of SVP among Austrian midwives and to identify potential prevention strategies to mitigate its effects. By addressing this gap, the study adds new context-specific insights to the existing body of knowledge and supports the development of targeted interventions in maternity care. We hypothesized that longer work experience and higher workloads increase the risk of becoming an SV. Furthermore, we hypothesized that midwives identifying as SVs prefer different support measures compared to those who have not experienced SVP. Regarding symptom severity, we hypothesized that certain personality traits and the type of incident leading to SVP, influence symptom severity. In an explorative approach, we also investigated whether the affected individuals received support is associated with symptom severity.

## METHODS

### Study design and data collection

Our study is a cross-sectional study conducted among midwives in Austria. We used the web-based SurveyMonkey platform (San Mateo, CA, USA) to administer the survey. Data collection took place between 16 October and 1 December 2023, conducted by the Austrian Second Victim Association in collaboration with the Austrian Midwives Association (ÖHG). To recruit participants, the Austrian Midwives Association sent an initial email containing study information and a survey link. As the vast majority of midwives in Austria are members of this association, eligibility criteria included membership in the Austrian Midwives Association and active employment as a midwife in Austria. Moreover, midwives in training were also eligible for participation. The only exclusion criterion was lack of consent to participate. No monetary incentives were provided for participation. Only fully completed questionnaires were included in the analyses. The required sample size was calculated before data collection and amounted to 334 participants. Given the calculated sample size and the number of responses we received, no further emails were sent to recruit additional participants.

To ensure data protection and participant anonymity, no personal data that could identify individual participants were collected. No cookies or IP addresses were stored, and the questionnaires were available only in anonymized form. Participants were informed of these measures before the survey began, and consent was obtained by requiring them to confirm their agreement in a designated field before proceeding with the survey. Ethical review and approval were waived for this study after the concept was presented to the ethical review board for the Viennese Hospitals and Vinzenz Holding before conducting the study.

### Survey instruments

To investigate the SVP among the participants of this study, we utilized the SeViD-questionnaire, described in detail elsewhere^17^. It consists of three domains and 40 items assessing general experience with the SVP, symptoms, and support measures. The first domain examines participants’ general experience with the SVP, including knowledge of the term, prevalence, incidence, triggering key events, received support, and self-perceived recovery time. The second domain assesses symptoms, with participants rating the severity of 20 possible symptoms on a three-point Likert scale (0=not pronounced at all, 1=weakly pronounced, 2=strongly pronounced). The third domain presents 13 potential support measures, which participants evaluate on a four-point Likert scale (1=not helpful at all, 2=rather not helpful, 3=rather helpful, 4=very helpful). Items in the symptoms domain were answered only by participants who had previously identified themselves as SVs, whereas all participants could respond to items in the support measures domain.

To assess the five personality dimensions – extraversion, openness, agreeableness, conscientiousness, and neuroticism – we used the BFI-10 questionnaire^18^. This questionnaire consists of 10 items, with two items measuring each personality trait. One item per trait is reverse-coded, requiring recoding to calculate the overall score.

### Statistical analysis

Demographic variables and applied instruments were summarized using descriptive statistics. Means and standard deviations (SD) were reported for interval-scaled data, while frequencies (n) and percentages (%) were used for nominal and ordinal variables. The analysis was conducted using SPSS Statistics Version 29 (IBM, New York, NY, USA). Statistical significance was defined as a p<0.05.

To test the first two hypotheses – that longer work experience and higher workload increase the risk of becoming an SV – we performed a binary logistic regression analysis. We chose longer work experience and higher workload as independent variables because previous studies have shown that both increase the likelihood of experiencing adverse events^2-4^. The outcome variable (SV: yes vs no) was dichotomized by grouping participants who had experienced SVP at least once into one category (SV: yes) and those who had not into a second category (SV: no). Age and years of work experience were included as independent variables to assess the influence of work experience on SVP occurrence. To operationalize workload, we used the number of women under care during pregnancy, including birth care, as an independent variable. Given the diverse professional backgrounds of midwives, we excluded variables that did not apply to all participants, such as bed capacity, since not all midwives worked in hospital settings. We also performed a binary logistic regression analysis to test the hypothesis that certain personality traits, the type of incident leading to SVP, and whether affected individuals received support influence symptom severity. We first calculated an overall sum score for the symptoms and then performed a median split to dichotomize the outcome variable. This resulted in the dependent variable (symptom severity) being categorized as high versus low overall symptom load. Additionally, the independent variable ‘support received’ was dichotomized (yes vs no).

Before performing both binary logistic regression analyses, we assessed whether the model met the necessary assumptions. These included a sufficient sample size of at least ten participants in each category of the binary dependent variables per independent variable, and the absence of multicollinearity^19^. The sample size was insufficient to include all planned independent variables. Before excluding variables from the model, we assessed multicollinearity. To do so, we examined the correlation matrix and the variance inflation factor (VIF), considering correlation values above 0.8 and VIF values above 5 as indicators of multicollinearity^20^. Multicollinearity was detected for the variables: age and work experience; therefore, age was excluded from both analyses. After this exclusion, the model met the first assumption. No adjustments were performed for both binary logistic regression models.

To evaluate model fit, explanatory power, and the ability to discriminate between binary outcomes, we calculated Nagelkerke’s pseudo-R² and assessed the area under the curve (AUC) of the receiver operating characteristic (ROC) curve^19^.

Since another hypothesis of the study was to compare ratings of possible support measures between SVs and non-SVs, we performed a Mann-Whitney U test for independent samples. To measure the effect size of significant differences between groups, we calculated^21^ Pearson’s correlation coefficient (r) using the equation r=z/√N.

Before analysis, the data were checked for missing values. Cases with a high number of systematically missing values (at least five consecutive missing responses in central questions) were excluded (171 in total)^22^. Isolated missing values were handled by listwise exclusion from the respective analyses^19^.

## RESULTS

Of the midwives contacted through the Austrian Midwives Association, 555 participated and 487 completed the survey in full. The mean completion time was about 10 minutes.

All survey participants were female, with a mean age of 39.2 years (SD=11.05, range: 18–65). Their work experience ranged from 1 to 44 years (mean=14.4, SD=11.49). Most midwives reported working part-time (n=278; 57.1%). The majority were employed in hospital settings (73.1%; n=356), followed by those who were self-employed without a contract with health insurance companies (45.2%, n=220). When asked about their primary area of work, most indicated the delivery room (77.0%; n=375).

Among midwives working in hospitals, the largest group was employed in facilities with a bed capacity between 100 and 299 (19.1%; n=93), followed by hospitals with over 600 beds (17.2%; n=84) and those with 300–599 beds (14.2%; n=69). The fewest worked in hospitals with <99 beds (8.6%; n=42). Regarding the number of annual births in their institutions, most reported 500–1000 births (24.8%; n=121), followed by 1000–2000 births (24.6%; n=120). The midwives surveyed cared for an average of 68.84 pregnant women annually (SD=31.92, range: 0–100).

When asked about the SVP, slightly more than half of the participants were familiar with the term (51.5%; n=251). After the term was explained, 80.5% (n=392) reported experiencing SVP more than once, while 13.8% (n=67) had experienced it once. Twenty-eight participants (5.7%) indicated that they had not experienced it. Thus, the overall prevalence of SVP among participants was 94.3%, with an incidence of 51.3% (n=250).

Table 1 provides a summary of the key triggering events. Participants who selected ‘other’ for their triggering event provided additional details in free-text responses (excerpts):

**Table 1:** Key events triggering SVP among Austrian midwives, SeViD-A3 cross-sectional study, 2025 (N=459)

*Key event*	*% (n)*
Incident without patient harm (near miss)	39.4 (181)
Incident with patient harm	19.0 (133)
Unexpected death or suicide of a patient	13.7 (63)
Aggressive behavior of a patient or their relative	11.3 (52)
Unexpected death or suicide of a colleague	1.5 (7)
Other	5.0 (23)

*‘Aggressive behavior by doctors towards patients’*

*‘Violence in the delivery room’*

*‘Uncollegial behavior’*

*‘Violent vaginal examination - physically no damage, but psychologically I’m not sure about this point, or rather I would say yes’*

*‘Physical and psychological violence in the delivery room, refusal of analgesia, racist behavior of staff’*

*‘Massive violation of a woman’s boundaries by a midwife’*

*‘Refugee woman who has obviously experienced violence and an aggressive gynecologist’*

**Figure 1:**
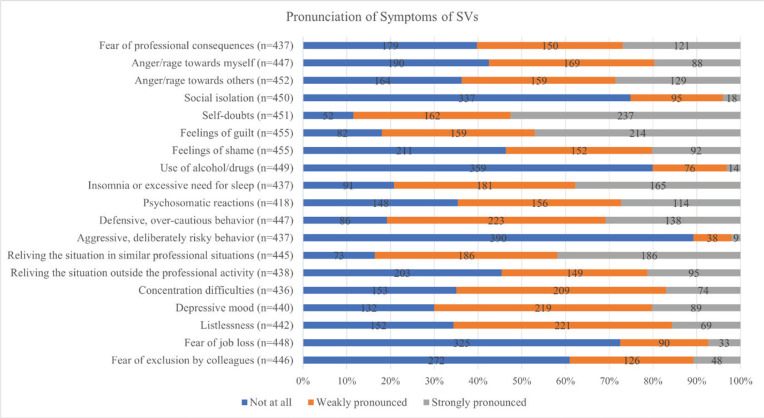
Symptoms of the midwives who identified as SVs, SeViD-A3 cross-sectional study, 2025

Most key events were unrelated to the COVID-19 pandemic (88.9%; n=433). Over half of the affected midwives received support from others (56.6%; n=260), while 35.5% did not receive help but had not sought it, and 7.8% (n=36) did not receive help despite requesting it. The vast majority turned to colleagues for support (94.2%; n=245), followed by family or friends (53.3%; n=139), superiors (36.2%; n=94), and professional counseling (21.5%; n=56). Only 5% (n=13) of those who received support sought it from upper management. Almost half of the midwives who have experienced SVP recovered from it within a month (48.9%, n=224). Eighty-six persons (17.7%) stated to not have fully recovered, yet. The most prominent symptoms among affected midwives were self-doubt (strongly pronounced in 52.5% of participants), feelings of guilt (strongly pronounced in 47.0%), and flashbacks in similar professional situations (strongly pronounced in 41.8%). The severity of all reported symptoms is shown in Figure 1.

**Figure 2:**
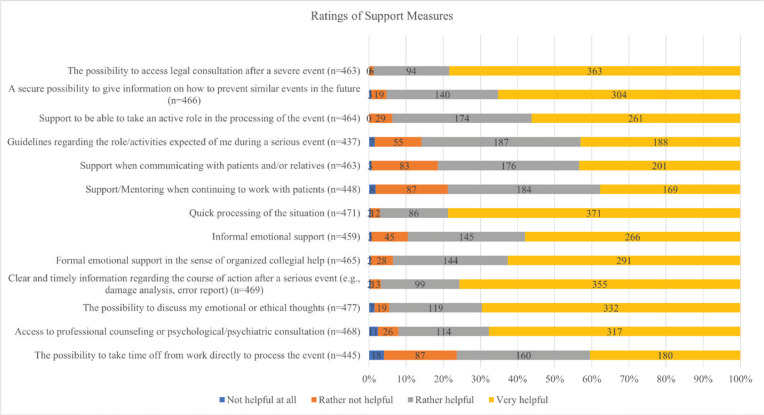
Ratings of possible support measures, SeViD-A3 cross-sectional study, 2025

When presented with different support measures, all were favorable to the participants of this study. Most favored were the possibility of a quick processing of the event (very helpful for 78.8%), the possibility to access legal consultation after a severe event (very helpful for 78.4%), and clear and timely information regarding the course of action after a serious event (very helpful for 75.7%). The ratings of all support measures are displayed in Figure 2.

The first binary logistic regression revealed that neither work experience nor higher workload significantly influenced the likelihood of experiencing SVP. Nagelkerke’s R²=0.013 and the AUC=0.587 indicate very low explanatory power and discriminative ability, suggesting poor model performance. Due to the absence of significant results, we rejected the hypothesis that greater work experience and higher workload increase the likelihood of experiencing SVP.

In the second binary logistic regression, events involving patient harm, support received, and neuroticism were significant predictors of high symptom load. Specifically, events with patient harm were associated with a 92% increased likelihood of high symptom load compared to incidents without patient harm (OR=1.92; 95% CI: 1.11–3.31, p=0.02). Our explorative approach showed that receiving support was linked to a nearly fivefold increased likelihood of high symptom load (OR=4.97; 95% CI: 1.46–16.93, p=0.01). Additionally, higher neuroticism scores significantly increased the likelihood of high symptom load, with each unit increase in neuroticism (measured by the BFI-10) raising the odds by 61.5% (OR=1.62; 95% CI: 1.24–2.11, p<0.001). All results of this binary logistic regression are shown in Table 2.

**Table 2:** Factors influencing overall symptom load (high vs low), results of binary logistic regression, SeViD-A3 cross-sectional study, 2025 (N=457)

*Predictor*	*Regression coefficient B*	*p*	OR*	*95% CI*	
				*Lower*	*Upper*
Event with patient harm^a^	0.65	0.02	1.92	1.11	3.31
Unexpected death/suicide of a patient^a^	-0.49	0.12	0.62	0.33	1.14
Unexpected death/suicide of a colleague^a^	-1.72	0.05	0.18	0.03	1.03
Aggressive behavior of patients/relatives^a^	0.28	0.45	1.32	0.64	2.75
Other type of key event^a^	-0.44	0.35	0.65	0.26	1.62
Support received^b^	1.60	0.01	4.97	1.46	16.93
Extraversion	-0.12	0.31	0.89	0.71	1.12
Agreeableness	-0.03	0.86	0.98	0.74	1.28
Conscientiousness	-0.31	0.07	0.73	0.52	1.03
Neuroticism	0.48	<0.01	1.62	1.24	2.11
Openness	0.12	0.28	1.13	0.91	1.40

a Reference category is ‘event without patient harm (near miss)’.

b Reference category is ‘no support’.

* OR=Exp(B).

Nagelkerke’s R²=0.16 and the AUC=0.70 suggest a moderate level of explanatory power and acceptable discriminative ability for this model.

Based on the results of the binary logistic regression analysis, we accepted the hypothesis that certain personality traits, the type of incident leading to SVP, and whether affected individuals received support, influence symptom severity.

The Mann-Whitney-U test revealed that midwives who identified as SVs rated the support measure ‘access to professional counseling’ as less helpful than those who did not identify as SVs (p=0.03, r=0.02). Therefore, we accepted the hypothesis that midwives identifying as SVs prefer different support measures compared to those who have not experienced SVP.

## DISCUSSION

This study investigated the Second Victim Phenomenon (SVP) among Austrian midwives, revealing a high prevalence of SVP, with 94.3% of participants reporting at least one experience. The most common triggering events included incidents without patient harm (39.4%) and events with patient harm (19.0%). Most key events were unrelated to the COVID-19 pandemic (88.9%; n=433). The most pronounced symptoms among affected midwives were self-doubt (52.5%), feelings of guilt (47.0%), and flashbacks in similar professional situations (41.8%). These findings are in line with results from similar studies and are broadly consistent with the international literature on SVP in healthcare settings. Comparable patterns regarding symptom burden, influencing factors, and the relevance of institutional support have been observed in studies conducted in other countries and professional groups^11-15^, suggesting that the phenomenon shows cross-contextual similarities.

The first binary logistic regression analysis found that work experience and workload did not significantly predict SVP occurrence, leading to the rejection of the corresponding hypothesis. However, the second analysis identified events involving patient harm, received support, and neuroticism as significant predictors of high symptom load. Events with patient harm were associated with a 92% increased likelihood of high symptom severity. Though counterintuitive, a nearly five-fold increase in symptom severity was associated with having received support, which will be discussed. Additionally, higher neuroticism scores significantly increased the likelihood of high symptom load.

Lastly, midwives who identified as SVs rated access to professional counseling as less helpful than those who did not experience SVP, supporting the hypothesis that SVs prefer different support measures.

At 94.3%, this sample exhibits the highest reported prevalence of the Second Victim Phenomenon (SVP) among medical professionals assessed using the SeViD questionnaire and other measurement tools^11-15^. One possible explanation is the high risk of adverse clinical events in obstetrics^1,23^. Additionally, midwives may develop a particularly strong emotional connection to the women and children they care for, making them more vulnerable to psychological distress following unexpected clinical events^24^.

The most significant triggering events identified in this study can all negatively impact patient safety. Notably, the most frequently reported event – incidents without patient harm – demonstrates that even when no physical harm occurs, midwives may still experience significant psychological distress. A qualitative study supports this finding, showing that adverse events in obstetrics, even without patient harm, can have long-term consequences for midwives, such as anxiety and shock^25^. Similar negative psychological effects have also been observed among healthcare professionals in other fields^26^.

Notably, the free-text responses regarding triggering events frequently described violence by medical personnel toward patients. Concerns about disrespect and abuse in obstetric care have been recognized for years. In 2014, the World Health Organization (WHO) issued a statement addressing the prevention and elimination of mistreatment during childbirth^27^. This statement emphasizes that women have the right to high-quality healthcare, free from discrimination and violence. Although the WHO statement was published ten years ago, obstetric violence remains widespread. A nationwide cross-sectional study in Spain involving over 17000 participants found that 38.3% of surveyed women had experienced obstetric violence as part of their healthcare^28^. Additionally, a systematic review highlighted that this issue receives little attention, particularly in industrialized countries, and that its link to traumatic birth experiences is often underestimated^29^. The findings of this study indicate that obstetric violence is also a significant issue in Austria. Furthermore, 88.9% of the triggering incidents were not related to the COVID-19 pandemic. Though the peripartum period in particular has been associated with massive restrictions and thus stress in times of the pandemic, we could not observe a relevant impact of the pandemic on the SVP among Austrian midwives^30^.

Most midwives who sought support after a triggering event turned to colleagues (94.2%), highlighting the crucial role of peer support in coping with SVP. The significance of colleague support is reflected in the three-stage intervention model for second victims, where peer support constitutes the first stage, and 60% of affected individuals find it sufficient to process their experience^31,32^. Additionally, most systematic support programs for second victims incorporate peer support as a key strategy to alleviate distress^33,34^.

The first binary logistic regression model showed that neither work experience nor workload influenced the occurrence of SVP. However, the low goodness-of-fit and discriminatory ability suggest that the model requires improvement. Due to the limited number of midwives who did not identify as SVs, it was not possible to include additional independent variables that might have enhanced the model’s overall explanatory power.

The second binary logistic regression model indicated that events with patient harm, received support, and higher neuroticism scores increased the likelihood of a higher overall symptom load. The association between events with patient harm and increased symptom severity may be linked to the strong emotional bond midwives develop with their patients^24^. The association between higher symptom load and received support seems counterintuitive at first but since we cannot be sure of the direction of the relationship of these two variables, it may be explained by the fact that greater symptom severity is generally linked to an increased willingness to seek treatment or support for mental health concerns^35^. The connection between higher scores in neuroticism have been linked to a higher symptom load of SVs in previous studies, as well^11,14^. Further, neuroticism is linked to lower resilience which might contribute to the increased likelihood of a higher symptom load^36^.

### Limitations

This study has several limitations. First, its cross-sectional design prevents the establishment of causal relationships between the identified factors and SVP. Second, the use of a convenience sample may introduce selection bias, potentially leading to an overestimation of effects. However, the SVP prevalence observed in this study aligns with previous SeViD studies, suggesting that our findings are consistent with existing evidence. Another limitation is social desirability bias, which may have influenced participant responses. Midwives in this study might have felt compelled to provide answers they perceived as socially acceptable or aligned with professional norms, particularly regarding admitting mistakes or reporting symptom severity. This could have led to underreporting of more sensitive issues, such as the mental health impact of SVP. The sample consisted exclusively of Austrian midwives, with the majority employed in hospital settings at the time of the survey. As a result, the generalizability of the findings to other professional groups or countries is limited. However, due to the comparison of our results with other studies, we are confident that generalizability is possible to some extent.

## CONCLUSIONS

The very high prevalence of the Second Victim Phenomenon (SVP) in this study underscores the urgent need for comprehensive and easily accessible support for midwives in Austria. Information campaigns as well as preventive and reactive measures should be developed and implemented. Given that midwives primarily seek support from colleagues, peer support programs should play a central role in intervention strategies.

As no significant workplace-related risk factors for SVP were identified, general support measures should be established. Special consideration must be given to the diverse work environments of midwives to ensure that self-employed midwives, who may lack team-based support, also benefit from these initiatives. Providing systematic and timely support could help retain midwives in the profession.

These findings highlight the importance of a structured approach to supporting midwives. Implementing targeted interventions with special emphasis on peer support as favorite source of support can not only enhance mental wel-lbeing among midwives but also contribute to improving patient safety and the overall quality of healthcare.

## Data Availability

The data supporting this research are available from the authors on reasonable request.
